# Modified Nanocellulose Hydrogels and Applications in Sensing Fields

**DOI:** 10.3390/gels11020140

**Published:** 2025-02-17

**Authors:** Lan Yang, Qian-Yu Yuan, Ching-Wen Lou, Ting-Ting Li, Jia-Horng Lin

**Affiliations:** 1 School of Textile Science and Engineering, Tiangong University, Tianjin 300387, China; lanyang202210@163.com (L.Y.); yuan_qianyu@163.com (Q.-Y.Y.); cwlou@asia.edu.tw (C.-W.L.); 2 Department of Bioinformatics and Medical Engineering, Asia University, Taichung City 413305, Taiwan; 3 Fujian Key Laboratory of Novel Functional Textile Fibers and Materials, Minjiang University, Fuzhou 350108, China; 4 Tianjin and Ministry of Education Key Laboratory for Advanced Textile Composite Materials, Tiangong University, Tianjin 300387, China; 5 Shaoxing Keqiao Institute, Tiangong University, Shaoxing 312030, China; 6 Advanced Medical Care and Protection Technology Research Center, Department of Fiber and Composite Materials, Feng Chia University, Taichung City 407102, Taiwan; 7 Advanced Medical Care and Protection Technology Research Center, College of Textile and Clothing, Qingdao University, Qingdao 266071, China

**Keywords:** cellulose-based hydrogel, cellulose modification, cellulose-based hydrogel modification, sensor, pressure sensing

## Abstract

Due to the intensification of global warming and the greenhouse effect, the exploration and research of sustainable sensors have become a research direction of people. Cellulose-based hydrogels, as a new kind of green material with strong plasticity, have become a popular material for sensor development. Due to the limited mechanical properties and poor compatibility of single-cellulose-based hydrogels, researchers have modified them to not only retain the original excellent properties of cellulose hydrogels, but also increase other properties, which has broadened the field of developing cellulose hydrogel sensors. From 2017 to 2020, cellulose-based hydrogel sensors were mainly used for biosensing applications, with a focus on the detection of biomolecules. Since then, researchers have increasingly turned their attention to pressure and strain sensors, especially those that are flexible and suitable for wearable devices. This paper introduces the modification of cellulose and cellulose-based hydrogels in detail, and lists the applications of modified cellulose-based hydrogels in different functional sensor directions, which provides different ideas for the application of modified cellulose-based hydrogels in the field of sensing, and proves that they have great potential in the field of sensing.

## 1. Introduction

With global warming, the issue of the greenhouse effect is escalating. The research and development of sensors that are beneficial to environmental sustainable development have become the key focus of recent studies. As a novel type of green material, cellulose-based hydrogels have gained increasing attention. Sensors based on nanocellulose hydrogels have more significant advantages than similar products in pressure and strain detection, pH detection, humidity detection and other aspects, have become the basis of a large class of innovative smart sensors, and are expected to develop more functions.

As the principal structural component of the plant cell wall and one of the most abundant polysaccharides in nature, cellulose has a carbon content exceeding 50% within the plant kingdom. Cellulose is insoluble in water and the majority of organic solvents, and is colorless and odorless. It possesses outstanding thermal stability, biocompatibility, biodegradability, regeneration and recyclability, and is extensively utilized in fields such as hydrogels and aerogels. Research into cellulose is predicated on people’s need for biocompatible products and environmentally friendly ones [1], so a variety of new materials based on cellulose are being gradually put into development, and cellulose is expected to become an important chemical resource in the future [2,3]. However, its poor chemical tolerance, limited mechanical strength, insufficient dimensional stability and poor compatibility with other materials are also problems that limit the use of cellulose. Researchers have discovered that modification serves as an effective strategy to assist cellulose in expanding its applications. The modification of cellulose is categorized into physical modification and chemical modification [4,5,6]. By adjusting the structure and properties of cellulose, modification can not only allow cellulose to retain its original performance advantages, but also enhance its other properties by adding different functional groups to meet the needs of various uses, so as to develop more possibilities [7,8]. For example, given the easy modification of nanocellulose surfaces, the surfaces can be treated to create more ideal multifunctional materials.

Hydrogels are three-dimensional network structures composed of hydrophilic polymers. They have the ability to absorb, swell and release large amounts of water and other biological fluids. They have excellent biocompatibility, environmental friendliness and pressure resistance. Based on their various raw materials, hydrogels can be divided into two types: natural sources and synthetic ones [9]. Hydrogels can be categorized into physical and chemical forms, in accordance with their crosslinking. Among these, physical hydrogels connect molecules through interionic interactions or hydrogen bonds, while chemical hydrogels form covalent bonds [10]. Due to these properties, hydrogels show broad application prospects in many fields, such as food processing, biomedicine, agricultural production, water quality treatment [11], sensing technology [12,13] and drug delivery [14,15].

The existence of hydrogen bonds and van der Waals forces endows cellulose or cellulose derivatives with a hydrophilic surface, enabling them to be crosslinked into a three-dimensional network of cellulose hydrogels with a multitude of different metals, organics and polymers through hydrogen, covalent or ionic interactions. Cellulosic hydrogels can retain large amounts of water or aqueous solutions, such as physiological solutions, without dissolving or losing their structural integrity, and are a combination of cellulosic polymers and water [16]. However, single-cellulosic hydrogels have defects, such as poor mechanical properties and single properties, so modifying other materials with cellulosic hydrogels has become one of the most effective ways to improve their properties. Modified cellulose-based hydrogels can optimize the properties of hydrogels, like self-adhesion, adsorption and mechanical strength [17].

**Figure 1:**
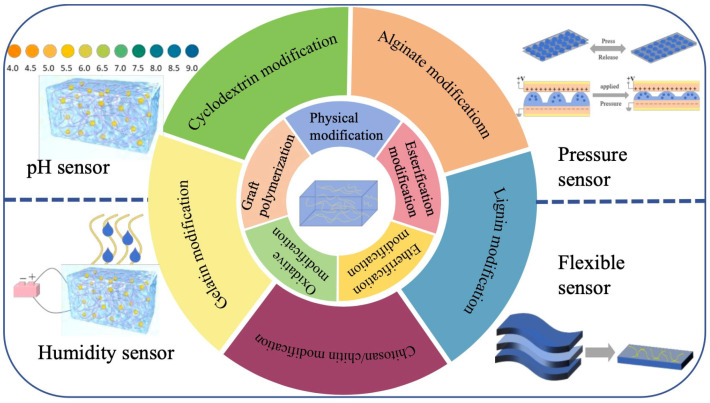
Modification method of modified cellulose-based hydrogel and its applications in sensors.

In this review, we present the physical and chemical modification of cellulose and categorize the prevalent materials that can be modified by cellulose hydrogels. Finally, we overview the applications of nanomodified cellulose hydrogels in various types of sensors. Figure 1 shows the modification method for modified cellulose-based hydrogel and its applications in sensors. It provides clues and ideas for further development of the functions of modified nanocellulose-based hydrogels, provides different solutions for the research of nanocellulose-based hydrogels in sensors, and demonstrates their potential in sustainable development.

## 2. Cellulose Modification

Although nanocellulose has received increasing attention from researchers due to its excellent physicochemical properties, its hydrophilicity has limited its application in hydrophilic or polar environments. Nanocellulose remains in a state of energy instability for a long time, which is due to the large diameter, high specific surface area and rich hydroxyl functional groups of nanocellulose, which provide a large number of active sites and high surface energy for the cellulose. Therefore, under the action of a large number of hydroxyl groups, it is easy for nanocellulose to aggregate with other surrounding nanocelluloses, reducing its surface energy and allowing it to reach a stable state, which seriously hinders the further application and development of nanocellulose [18]. Therefore, various physical and chemical approaches are employed to modify the hydroxyl group on the surface of nanocellulose. Among them, chemical modification is the most significant method, including esterification, oxidation, etherification and grafting copolymerization reactions, which typically involve the alteration of the hydroxyl group in the structure of nanocellulose and endow it with specific functions [19]. Through modification, a series of ionic groups can be introduced to enhance the hydrophilicity of cellulose. The crystallinity and polymerization degree of modified cellulose are significantly decreased, and its physical and chemical properties are significantly improved, compared to those of unmodified cellulose, showing more remarkable advantages, which can also make cellulose more appropriate for more complex application scenarios.

### 2.1. Physical Modification of Cellulose

Physical modification of cellulose is a form of treatment that does not involve changes in chemical composition or chemical reactions. It uses applied physical, mechanical or physicochemical means, such as mechanical crushing, swelling, compounding, surface adsorption and other operations, to adjust the structure and form of cellulose, such as by thin film, micro-powder, spheroidization and nano methods. This modification is capable of maintaining the original chemical structure of cellulose and enhancing its surface properties, thus enabling it to acquire new properties and functions. At present, liquid ammonia treatment [20,21], high-pressure vapor flash, ultrasound and plasma treatment are several commonly used modification techniques.

**Figure 2:**
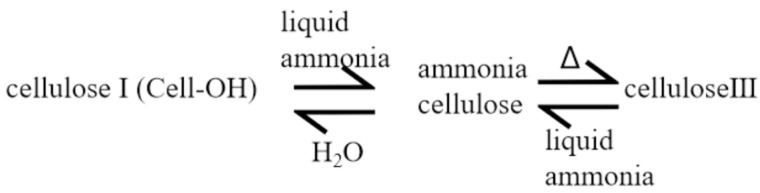
Liquid ammonia treatment of cellulose.

Liquid ammonia treatment is one modification method used for cellulose [22]. After being treated with liquid ammonia, considering that the density, viscosity and surface tension of liquid ammonia are much lower than that of water, it is very easy to penetrate into the interior of the fiber, or even the middle region in the lateral order. This penetration gives rise to the expansion of the cellulose structure, thereby disrupting the hydrogen bond network within the cellulose, enhancing the activity of the molecular chains and reducing the orientation of the fiber macromolecules. The fiber lattice will expand and disintegrate to a certain extent, eventually leading to a decrease in fiber crystallinity and the formation of an easily decomposed cellulose–ammonia complex. Ammonia-modified cellulose can be obtained by volatilization of ammonia gas. The swelling degree of cellulose fiber is relatively low, and the decrease in the crystallinity of cellulose fiber after liquid ammonia treatment is smaller than that after concentrated alkali treatment. The larger the pore size is, the smaller the number of micropores becomes. The internal micropores shift to a smaller pore size, and the pore size is small and evenly distributed. Therefore, liquid ammonia treatment makes cellulose molecules expand through physical and chemical action, thus changing the aggregation state, morphological structure and performance of cotton fibers. Figure 2 shows the reaction formula for liquid ammonia treatment of cellulose.

Zhu et al. [23] conducted a comparison between cotton fiber treated with liquid ammonia and untreated cotton fiber, and found that the cotton fiber treated with liquid ammonia displayed expansion, a smooth longitudinal surface and fewer cracks. At the same time, the amorphous region in the cotton fiber expanded, and some crystal structure was damaged, resulting in a decrease in fiber crystallinity. The liquid ammonia treatment improved the fiber’s breaking strength and elongation at break, and increased the crease recovery angle. To investigate the influence of liquid ammonia treatment on ramie fiber and fabric, Zhang et al. [24] discovered that liquid ammonia treatment expanded the non-static region of the fiber, weakened part of the crystal structure and further reduced the crystallinity of the fiber. Similarly to cotton fiber, the crease recovery rate, tensile strength and softness of treated ramie fabric are stronger than those of untreated fabric, even exceeding the effect of alkali-treated fiber [25].

High-pressure steam flash is another method of fiber modification [26]. Following a long period of soaking in water and deep immersion, the cellulose is placed in a closed container for high-temperature processing. In this process, the resulting water vapor creates a high-pressure environment, followed by a rapid drop to normal pressure for a set period of time, which causes the supramolecular structure of the cellulose to be damaged and the hydrogen bond within the molecule to be broken. This method can immediately exhaust the high-temperature hot steam that penetrates into the cellulose material, so that the supramolecular structure of the cellulose is destroyed. This method has the characteristics of low cost and effective collection of hemicellulose. Chen et al. [27] used high-pressure flash technology to treat wheatgrass, making the hemicellulose and part of the lignin in wheatgrass degrade into low-molecular matter, and making the fiber loose and porous, making it easier to burn. The results of XPS analysis demonstrated that the surface area and the amount of hydrophilic groups of the fibers increased after steam explosion treatment, thereby improving the hydrophilic properties of the fibers.

As a very effective technology for the preparation of nanomaterials, ultrasonic treatment has the advantages of simple operation, easy control and high efficiency, and is widely used to assist the preparation of nanomaterials [28]. By breaking down the anti-depolymerization barrier to extract cellulose with the help of chemical agents, tiny bubbles in the liquid form, grow and eventually collapse as the sound waves compress and disperse. During this procedure, the implosion of the bubble can generate a considerable amount of energy, resulting in local high temperature. This breaks the hydrogen bonds among the swelling fibers, thereby enabling the separation of microfibril and even base fibrillar fibers. This is beneficial for the dissolution of cellulose or its structural modification, and subsequently, cellulose nanofibers (CNFs) can be acquired. Lu et al. [29] used an ultrasonic-assisted acid method to prepare potato residue nanocellulose. With increasing ultrasonic power, the yield of potato residue nanocellulose gradually ascends, mainly due to the cavitation effect, thermal effect and mechanical effect of the ultrasonic wave, which enables acid to more easily penetrate into the interior of cellulose and react.

Plasma treatment is likewise an efficient approach for modifying cellulose [30]. Plasma refers to a partially ionized gas that has approximately equivalent positive and negative charges, along with density. It is composed of six types of active particles, namely electrons, positive ions, negative ions, ground-state atoms or molecules, excited-state atoms or molecules, and free radicals [31]. Based on the working gas employed, plasma treatment can lead to ablation, activation or crosslinking of the material. These procedures can alter numerous surface characteristics of the polymer, such as wettability, adhesion, dyeing capacity, refractive index, chemical stability, lubrication properties and biocompatibility. During the plasma treatment, parts of the polymer molecular chain break, thus forming new functional groups. These newly formed functional groups can be used not only as reaction sites for subsequent chemical modifications, but also as fixed points for polymer grafting.

Kola’r et al. [32] employed plasma discharge technology to modify cellulose fibers, and elaborately characterized the properties of the modified cellulose fibers using XPS, FTIR and SEM. It was discovered that plasma treatment led to the degradation and ablation of the fiber surface. Moreover, the surface morphology and width of the fibers were also altered. During the treatment process, the surface of the fiber was oxidized, and the structure of the D-glucose ring was impaired. Initially, hydrophilic fibers (whether raw materials or plasma-treated samples) gradually change into hydrophobic fibers within 24 h after plasma treatment.

### 2.2. Chemical Modification of Cellulose

Chemical modification is mainly achieved through reactions related to the hydroxyl group of cellulose. It is based on the large number of hydroxyl groups in the cellulose chain, which can be used to achieve functionalization with various organic compounds, according to demand. Non-polar or hydrophobic groups can be introduced to enhance their interfacial compatibility in the composite. Chemical modification is also capable of endowing the surface of cellulose with either a positive or negative charge, enhancing the dispersion of cellulose in solvents. The chemical modification techniques of cellulose comprise acid–base hydrolysis, esterification, sulfonation, substitution reactions and the like [33]. By means of these methods, the physical characteristics of cellulose can be changed and its application scope can be expanded. The types and applications of chemically modified cellulose are shown in Table 1.

#### 2.2.1. Etherification Modification

The etherification modification of cellulose is a chemical reaction through the alcohol hydroxyl group of cellulose in an alkaline environment. After etherification, cellulose can be dissolved in water, dilute lye and organic solvents, and exhibits thermoplastic properties [34]. Its solubility is mainly influenced by three aspects: Firstly, the properties of the introduced groups. The larger the volume of the groups becomes, the lower the solubility of the cellulose ether becomes; the stronger the polarity of the groups is, the better the solubility of the cellulose ether in water will become. Secondly, the substitution degree and distribution of etherification groups on macromolecular chains. Thirdly, the degree of polymerization of the cellulose ether. The higher the degree of polymerization is, the more difficult the dissolution will be. Conversely, when the degree of polymerization is low, a higher degree of substitution can enable the cellulose to be soluble in water. On account of its variety and superior performance, cellulose ether has been widely used in many industries, such as construction, cement, petroleum, food processing, textiles, cleaning supplies, coatings, medicine, paper manufacturing and electronic components [35].

Wang et al. [36] modified microcrystalline cellulose (MCC) by cationic etherification, and characterized the modified cellulose. The results showed that the basic chemical structure of the modified microcrystalline cellulose (MD-MCC) was not damaged, but its surface showed a porous structure, and the crystallinity and thermal stability were slightly reduced. The addition of MD-MCC to the starch membrane provided better dispersibility and compatibility than the addition of MCC alone. The tensile strength, light transmission and water vapor barrier properties of the starch–MD-MCC composite membrane were superior to those of the starch–MCC composite membrane.

Oyewo et al. [37] modified cellulose nanocrystals (CNCs) derived from wood chips, mixed them with NaNO_2_, and added NaHCO_3_ to generate anionic oxygen groups on the CNC surface. The modified CNCs showed high porosity, uniform particle size distribution and a needle shape. This process reduced the zeta potential of CNCs. By detecting the variation in metal ion concentration in aqueous solution, and in conjunction with EDS and XPS analysis, the effective removal capacity of CNCs for metal ions was verified.

In order to recover hydroxypropyl cellulose (HPC), Joshi et al. [38] used alkalization and etherification processes to extract hydroxypropyl cellulose from waste paper. HPC was isolated from the mixture by treating the alkaline cellulose with excess propylene oxide, followed by a post-reaction process of cooling, acetic acid neutralization and acetone precipitation. A new recycling route for waste paper was explored, and proved that waste paper can be used as a promising raw material for HPC synthesis.

#### 2.2.2. Oxidative Modification

The oxidative modification of cellulose refers to the partial oxidation of cellulose. Since there are three hydroxyl groups on cellulose glucoside, cellulose can be oxidized by different types of oxidants to produce various cellulose derivatives, and many active groups, such as aldehyde groups, ketone groups, carboxyl groups or enol groups, can be introduced to generate oxide materials with different properties [39]. Oxidative modification can be classified into selective oxidation and non-selective oxidation. The oxidation of cellulosic materials holds a central position in cellulosic chemistry, and this procedure can endow each cellulosic material with distinctive properties. The oxidation modification of cellulose to generate high-added-value products has turned into a key factor influencing the overall and chemical properties of cellulosic materials [40]. In addition, the preparation of oxidized cellulose containing a carboxyl group has some useful medical application value, so it has special application value. Oxidized cellulose is capable of being bioabsorbed and easily degraded under physiological circumstances, and is widely employed as an absorbable hemostatic stent material [41], as well as a layer for preventing postoperative adhesion [42]. In addition, oxidized cellulose is also widely used in agriculture, cosmetics and medicine.

Li et al. [43] developed an efficient method for preparing CNF. In this method, coniferous-wood-bleached sulfate pulp fiber is used as an independent microreactor to achieve effective in situ oxidation of cellulose chains, thus promoting the nanoization of fibers in subsequent mechanical treatment. By introducing hydrogen peroxide, the C2, C3 and C6-hydroxyl groups on the cellulose chain are oxidized to carboxyl groups, which augments the electrostatic repulsion force among the fibers and eases the separation of micro- and nanofibers. Furthermore, the 1, 4-β-D-glucoside bond of the cellulose chain is disrupted through oxidation, leading to a considerable reduction in the degree of polymerization of cellulose macromolecules.

Saito et al. [44] used sodium hypochlorite and catalyzed amounts of 2, 2, 6, 6-tetramethylpiperidin-1-oxy radical and sodium bromide to oxidize natural cellulose suspended in water to varying degrees. The results showed that an aldehyde group of up to 0.225 mmol/g was introduced into the natural cellulose fibers through rhythm-mediated oxidation, which remained stable and improved the wet strength of the natural cellulose.

In addition, studies by Azzam et al. [45] indicate that the oxidation of CNCs with high-acid salts increases the degree of oxidation, and that almost all of the resulting carbon groups are converted to hemiacetals by recombination with neighboring hydroxyl groups. Periodate oxidation and reduction amination are common methods for adjusting the chemical properties and morphology of CNCs.

#### 2.2.3. Esterification Modification

**Figure 3:**
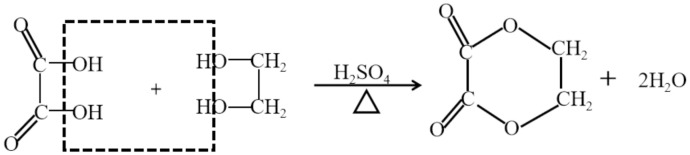
Structural formula of esterified modified cellulose.

Esterification and etherification have a significant role in the derivatization of cellulose. The hydroxyl group present on the cellulose molecular chain has the ability to react with acids, anhydrides, acyl halides and other substances to generate esters, and it can also react with alkyl groups to form cellulose ethers. The structural formula of esterified modified cellulose is shown in Figure 3. In the 1950s and 1960s, the industrial application of these reactions was gradually realized. Among cellulose ester products, cellulose nitrate ester, cellulose acetate and cellulose xanthate are the most common and important ones, which have been widely used in many industrial fields and scientific research directions, such as coatings, cosmetics, medicine, textiles, plastics, tobacco, adhesives and film science.

Sakovich et al. [46] prepared cellulose nitrate, corresponding to the basic properties of high-viscosity paint-grade bakelite, by esterification of cotton cellulose pulp from a rapidly renewable raw material—agro-industrial waste—, oat husk or a mixture of sulfuric acid and nitric acid. The prepared cellulose nitrate had good mechanical sensitivity, good chemical compatibility and high chemical stability when mixed with plasticizers.

Ramirez et al. [47] put forward a straightforward method for the surface esterification of CNCs. The CNCs they obtained from microcrystalline cellulose were acetylated with citric acid, which is the alpha-hydroxy acid found in citrus fruits, in a medium containing sucrose or glucose from some mold. The chemical structure, crystallinity, morphology, thermal decomposition and dispersion of acetylated CNCs in non-polar solvents were characterized. The results showed that the scheme is suitable for simple surface acetylation of CNCs.

#### 2.2.4. Grafting Polymerization

Cellulose, with its hydroxyl group as the grafting point, can link various polymers to its main chain to achieve grafting polymerization modification. Given the structural features, functional attributes and variations in molecular weight of grafted polymers, various properties and applications can be achieved for cellulose. The introduction of macromolecules can optimize the properties of cellulose without completely undermining the inherent advantages of cellulose materials. Depending on the polymerization conditions, the length of the branch chain or graft chain will also vary accordingly. There are many kinds of monomers that can be grafted, among which propylene and vinyl monomers are the most widely used and have become the research hotspots of the chemical modification of cellulose. The traditional grafting method is to carry out multi-phase grafting copolymerization between cellulose and acrylic acid, acrylonitrile, methyl methacrylate, acrylamide, styrene and other polymer monomers to realize the multifunctionality of cellulose. Therefore, this method is widely used in biodegradable plastics, ion exchange resins, water-absorbent resins, composite materials, flocculants and chelating fibers.

Jiang et al. [48] used propylene ethyl trimethylchloride and ammonium cerium nitrate as initiators to modify the surface of CNCs by grafting polymerization, and successfully realized the combination of polypropylene ethyl trimethylammonium chloride polymer and CNCs. This process significantly increased the positive charge density of the CNCs’ surface, which greatly improved their adsorption capacity for anionic dyes.

On the other hand, Garcia-Valdez et al. [49] grafted three polymers (dimethylaminoethyl methacrylate), poly (diethyl methacrylate) and poly (diisopropyl aminoethyl methacrylate) onto the CNC surface through free radical polymerization technology. This gave the CNC surface the ability to respond to changes in carbon dioxide, forming a new type of nanocomposite material.

In addition, Xing et al. [50] managed to graft methylpropenoxy-benzyl dimethyl ammonium chloride onto cellulose fibers by means of a thio-carbonate-H_2_O_2_ REDOX system to obtain fibers with antimicrobial properties. The outcomes of FTIR and SEM indicated that the modified cellulose fibers were capable of effectively damaging the bacterial cell membrane, causing the aggregation or splitting of bacteria into fragments. It was found that the modified cellulose fibers showed a remarkable killing effect on *Escherichia coli*, which may allow them to become a new water treatment technology.

**Table 1:** Study of types and applications of chemically modified cellulose.

Chemically Modified Types	Cellulose Type	Method of Modification	Ref.
Etherification modification	Microcrystalline cellulose	Cationic etherification modification with NAOH.	[36]
Etherification modification	Cellulose nanocrystals	The addition of NaNO_2_ to an aqueous solution causes the cellulose to deprotonate, forming a sodium salt. Step 2: Add NaHCO_3_ to introduce the carbonic acid group, which opens through the bond to produce the anionic oxygen group on the surface of the material.	[37]
Etherification modification	Hydroxypropyl cellulose	Etherification of alkali cellulose with propylene oxide.	[38]
Oxidative modification	Cellulose fibers	Ferrous ions are preloaded into the fiber cell wall, and after the introduction of hydrogen peroxide, the catalytic oxidation of cellulose is initiated in the fiber cell wall structure.	[43]
Oxidative modification	Natural cellulose	Natural cellulose is oxidized using sodium hypochlorite and different catalytic amounts of tetramethylpiperidin-1-oxy radical and sodium bromide.	[44]
Oxidative modification	Cellulose nanocrystals	Periodate is used to oxidize cellulose nanocrystals.	[45]
Esterification modification	Cotton cellulose	Cotton cellulose is esterified using a mixture of sulfuric and nitric acid.	[46]
Esterification modification	Cellulose nanocrystals	Cellulose nanocrystals are esterified using citric acid as catalyst and acetic anhydride as a reagent and reaction medium.	[47]
Grafting polymerization	Cellulose nanocrystals	Acrylyl ethyl trimethyl chloride is grafted with ammonium cerium nitrate through initiator polymerization.	[48]
Grafting polymerization	Cellulose nanocrystals	Poly (dimethylaminoethyl methacrylate), poly (diethyl methacrylate) and poly (diisopropylaminoethyl methacrylate) are grafted onto cellulose’s surface.	[49]
Grafting polymerization	Cellulose fibers	The thiocarbon H_2_O_2_ REDOX system grafts methylacryloyl hydroxybenzyl dimethyl ammonium chloride onto cellulose fibers.	[50]

## 3. Modified Cellulose Hydrogel

Owing to the inadequate mechanical properties and limited functionalities of single-cellulose hydrogels, combining them with other materials to form composite hydrogels has emerged as an effective strategy to enhance their performance. Biomass, the most abundant substance on Earth, encompasses all animals, plants and microorganisms, and the various organic compounds derived, excreted or metabolized by these organisms. Research has shown that the unique structure and active groups of biomass, including amino groups, cyclic oligosaccharides and rigid aromatic rings, can optimize the properties of hydrogels, such as self-adhesion, adsorption and mechanical strength. For a detailed overview of the types and applications of biomass-modified cellulose hydrogels, refer to Table 2.

### 3.1. Cyclodextrin

Cyclodextrin (CD) is a series of cyclic oligosaccharides, formed from amylose catalyzed by CD glucosyltransferase produced by *Bacillus*, usually consisting of 6 to 12 D-glucose units. Among the many widely studied and useful CDs, the structures containing six, seven and eight glucose units are named alpha-, beta-, and gamma-CDs, respectively. Based on X-ray crystallography, infrared spectroscopy and nuclear magnetic resonance analysis, it can be seen that each of the D(+) -glucopyrane rings forming the CD molecule has a chair configuration. Each glucose unit is linked by a 1, 4-glucoside bond to form a ring. As the glucoside bonds that hold the glucose units together do not rotate freely, CDs are not cylindrical molecules, but rather slightly tapered rings. CDs are the ideal host molecule for enzymes discovered hitherto, and they themselves possess the properties of an enzyme model. Hence, CDs have received significant attention, and have been extensively utilized in the domains of catalysis, separation, food and medicine. Owing to the solubility and inclusion capacity of CDs in water, altering the physical and chemical properties of CDs has become one of the significant aims for the chemical modification of CDs [51].

Zhang et al. [52] investigated in situ crosslinked hydrogel dressings based on cellulose and modified CDs. Hydrogels were produced via the DMAc/LiCl (N, N-dimethylacetamide/lithium chloride) dissolution system by the formation of hydrogen and ester bonds among the hydroxyl group of cellulose, the hydroxyl group of modified CD and the catechol of catechins. The modified cellulose hydrogels have obvious interpenetration, remarkable porosity and controlled drug release characteristics, which can promote the rapid healing and regeneration of diabetic skin tissue, and give them great potential and value in wound treatment.

In addition, Sun et al. [51] developed a novel curable hydrogel composed of visible-solidified glycol chitosan and curcumin, with an inclusion compound formed by beta-CD. Through the continuous control of the release of curcumin, the hydrogel speeds up wound healing. Studies have indicated that this cellulose-based hydrogel has the potential to serve as a wet dressing to assist in facilitating the soft tissue repair of open fractures.

Liu et al. [53] used bamboo shoot cellulose as raw material, obtained carboxymethyl cellulose (CMC) through chemical modification, and used epichlorohydrin as a crosslinking agent to prepare a composite hydrogel with β-CD. In addition, Sun et al. developed an innovative curable hydrogel consisting of visible light curable glycol chitosan and curcumin, in which the inclusion compound was formed by β-CD. By continually regulating the release of curcumin, the hydrogel speeds up the healing process. The study found that this cellulose-based hydrogel has the potential to be used as a wet dressing to help promote the repair of soft tissue in open fractures.

Xia et al. [54] prepared a natural hydrogel film using CMC, CNCs and hydroxypropyl β-CD (HP-β-CD) as raw materials, and citric acid as a crosslinking agent, for the controlled release of neorhesperidin-copper. The hydrogel film has good cytocompatibility and no cytotoxicity, and can be used as a material for drug delivery and controlled release. The cellulose hydrogels modified by CD have good adsorption and slow release properties through coating, but their function is relatively simple. Host–guest interaction can also be used to prepare responsive shape-memory cellulose hydrogels, and reversible crosslinking can provide deformation power for the hydrogels.

### 3.2. Alginate

Alginate [55] belongs to the salt family of alginate. It is a long chain polymer of (1→4) -β-crosslinked D-manuronic acid and (1→4) -α-crosslinked gulonuronic acid, with a relative molecular mass of about 10^6^. It is mainly found in the cell wall and intercellular mucilage of brown algae, but also in some bacteria, such as Pseudomonas and azotobacter, that produce mucinous capsules. Under the action of dihydrazide adipate, polyethylene glycol diamine, lysine and other crosslinking agents, stable covalent crosslinked hydrogels can be obtained through a dehydration condensation reaction of amino and carboxyl groups. Alginate can also form ion bridges or chelation in the presence of Ca^2+^, and then form calcium alginate gel, which is a thermal irreversible gel; this gives it a very significant advantage compared with other colloids.

Alginate extracted from algae is insoluble in water, whereas sodium salts are soluble in water. The alginate substructure usually consists of three parts: the “M region” (mainly composed of manuronic acid), the “G region” (mainly composed of gururonic acid) and the “MG region” (containing both manuronic acid and gururonic acid). Ca^2+^ and other bivalent cations tend to bind to the G region, so calcium alginate gels are thought to be a three-dimensional network of molecules, with the G region of long chain molecules crosslinked by Ca. The composition of alginate varies from species to species, and the differences in its M/G value will significantly affect its physicochemical properties. Azotobacter vinelandii (Azotobacter vinelandii) is also capable of producing alginate, and has the potential to become a new source of alginate due to its ability to produce it in an artificial environment, irrespective of geographical and seasonal factors.

Silva et al. [56] achieved synchronous controlled release of furazolidone and bismuth through the use of a mixed hydrogel of sodium alginate and CMC. Detailed characterization of the hydrogel revealed that bismuth-containing samples exhibited higher resistance to degradation under neutral conditions, and their drug-controlled release performance was better than that of bismuth-free samples.

In addition, the preparation of pH-sensitive alginate–methylcellulose mixture hydrogel beads by a single water-in-water emulsion gel method is another alginate-modified cellulose hydrogel solution. Banerjee et al. [57] analyzed these modified hydrogel drug beads, and the results showed that the trivalent ion crosslinked beads not only increased drug embedding efficiency, but also strengthened drug release in an alkaline medium.

Wang et al. [58] successfully designed and fabricated an injectable mixed hydrogel containing alginate, gelatin and nanocrystalline cellulose by using an alginate–gelatin interpenetrating network, alginate ion crosslinking and supramolecular interaction. The mixed hydrogel showed moderate swelling characteristics during the molding process, had ideal mechanical properties and proved to have good biocompatibility.

### 3.3. Lignin

Lignin [59] is a kind of complex organic polymer. It is the second most abundant biomass resource in the plant kingdom after cellulose. It serves as an important structural material in the supporting tissues of vascular plants and some algae. Given that lignin is mainly located among cellulose fibers, it plays a role in withstanding pressure [60]. Therefore, the strength and toughness of the hydrogel system can be enhanced by lignin modification. From a chemical point of view, lignin can be regarded as a crosslinked phenolic polymer. The biopolymer consists of three phenylpropane units which are linked by ether and carbon–carbon bonds, forming a three-dimensional network structure. Lignin is rich in aromatic rings, fatty hydroxyl groups, aromatic hydroxyl groups and quinone groups.

Chen et al. [61] developed a novel lignin-grafted polyacrylamide/hydroxypropyl cellulose hydrogel with a semi-interpenetrating polymer network structure whose conductivity exceeds that of other MXene-dependent polyacrylamide-based hydrogels [62]. It also has unique adhesion, stretchability and antimicrobial properties, and can be used as an ideal flexible strain sensor. 

In addition, Park et al. [60] co-dissolved cellulose and lignin in 1-ethyl-3-methylimidazole acetate, and subsequently successfully fabricated cellulose/lignin composite hydrogel beads by reconstituting them with distilled water. They also fixed candida lipases to different types of cellulose/lignin hydrogel beads. The results indicated that the hydrogel is highly biocompatible, biodegradable and controllable, and might offer numerous potential applications in biocatalysis, biomedicine and bioelectronics.

In addition, due to the antibacterial properties of lignin and lignin-derived compounds, many researchers will use it as a wound healing agent in the form of dressings, to help patients repair skin and relieve pain. Danica et al. [63] developed a highly effective antibacterial material by combining bacterial cellulose with needinol dehydrogenation polymer to form a composite hydrogel specifically designed for the treatment of chronic wounds. The hydrogel can fight specific pathogenic microorganisms, showing an inhibition or killing effect. Further studies using high-performance liquid chromatograph–mass spectrometry have revealed that needinol dehydrogenation polymer oligomers released from the composite hydrogel may be the key component that gives it antibacterial properties.

Lu et al. [64] prepared a pH-responsive hydrogel using lignin and cellulose nanofibril, whose elastic modulus increased as the pH increased from 4 to 7, and gradually decreased as the pH increased further from 7 to 9. This is a direct result of the different stress–relaxation kinetics determined by the dynamic hydrogen bonding and swelling/contraction of the lignin nanoparticles.

### 3.4. Chitin/Chitosan

Chitosan is the result of the partial deacetylation of the natural polysaccharide chitin. It has a similar chemical structure to chitin and cellulose. Specifically, cellulose has a hydroxyl group at the C2 position, whereas chitin and chitosan have acetyl and amino groups, respectively, at the same position. These compounds are not only biodegradable, but also exhibit excellent cellular compatibility and a variety of biological activities, including, but not limited to, non-toxicity, antimicrobial action, anticancer effects, the reduction of lipid levels, and the enhancement of immune system function. In particular, because chitosan molecules contain free amino groups, chitosan is the only natural polysaccharide that is alkaline, and it is sometimes referred to as animal cellulose. The substance has a wide range of applications, including additives in the food industry, the textile industry, agriculture, environmental protection, beauty and skin care products, antibacterial materials, medical fibers, trauma care products, artificial tissue materials, slow-release systems of drugs, gene delivery technology, biomedical research, absorbable medical devices, tissue engineering technology materials, pharmaceutical product development and other fields, as well as the daily chemical industry.

Shang et al. [65] mixed two natural polyelectrolyte solutions, chitosan and CMC, and crosslinked them with glutaraldehyde to make amphoteric hydrogel membranes. The bending of the film under an electric field in different electrolyte solutions was investigated. This bending of the film in various electrolyte solutions allows it to be applied to microsensors and brakes, especially in the biomedical field.

In addition, Yan et al. [66] also used methylene bisacrylamide as a crosslinking agent and synthesized chitosan and CMC interpolymer complexes by irradiation in acetic acid/aqueous solution. The structures were analyzed through FTIR and atomic force microscopy. It was discovered that the hydrogel can expand in water, and its expansion characteristics are significantly influenced by the concentration of ions in the solution, which indicates that the material is a polyamphoteric hydrogel.

Berton et al. [67] prepared composite hydrogels using ionic liquid 1-ethyl-3-methylimidazole acetate to extract chitin- and cellulose-rich materials from shrimp shells and poplar. The composite hydrogels have improved mechanical properties, high porosity, water absorption and oxidation resistance, and can be used in tissue engineering scaffolds.

The amino group in the molecular structure of chitosan is more reactive than the acetyl amino group in the chitin molecule, resulting in the polysaccharide having outstanding biological functions and being able to undergo chemical modification reactions. Therefore, chitosan is considered as a functional biomaterial with greater application potential compared to cellulose [68].

### 3.5. Gelatin

Gelatin [69] is a macromolecular hydrophilic colloid formed through the partial hydrolysis of collagen. Its primary source is the breakdown of collagen in animal skin, muscle membrane and other connective tissues. Possessing amino and carboxyl groups, gelatin is a hydrophilic linear polymer with a protein structure, and has excellent biocompatibility and degradability. After degradation, non-toxic products can be formed and discharged from the body. According to its different uses, the quality requirements for gelatin are also different. When used as a binder, the main requirement is bond strength. When used in photography, food, medicine and other fields, emphasis is placed on the purity of the product. The molecular structure of collagen is a helix formed by three strands of polypeptide chains interwoven with each other. After treatment, the helical structure breaks down into different components: a single polypeptide chain (referred to as the alpha-chain), a β-component composed of two α-chains and a γ-component consisting of three α-chains. In addition, the length of these molecular segments can be shorter than the α-component or longer than the γ-component. It can be seen that gelatin is a polydisperse system with a certain molecular weight distribution, and its molecular weight distribution is different due to different process conditions, which has an impact on the physicochemical properties of gelatin. Gelatin is the product of collagen denaturation; belonging to the category of protein macromolecules, it has similar characteristics to those of protein macromolecules, but because of the particularity of its molecular structure, its physical and chemical properties are unique.

Kajjari et al. [70] successfully produced microspheres of semi-interpenetrating polymer network hydrogels of gelatin and hydroxyethyl cellulose by means of water-in-oil emulsion technology, and explored their application in the controlled release of anti-theylline. By analyzing the ratio of gelatin to hydroxyethyl cellulose in the mixture, the amount of the crosslinking agent added and the percentage of drug loading, they obtained a drug release curve. The results indicated that the physical state of the drug in the polymer hydrogel can remain stable.

In addition, Treesuppharat et al. [71] prepared hydrogel composites with excellent properties by copolymerizing bacterial cellulose with gelatin. These materials have outstanding thermal stability, chemical stability and mechanical properties, and are suitable for drug delivery systems.

**Table 2:** Modification types and applications of cellulose-based hydrogels.

Biomass	Method	Cellulose	Ref.
Cyclodextrin	In situ crosslinking using epichlorohydrin as a crosslinker	Bamboo shoot cellulose	[53]
Cyclodextrin	In situ crosslinking using citric acid as a crosslinker	Carboxymethyl cellulose	[54]
Alginate	The single water-in-water emulsion gel method	Methylcellulose	[57]
Alginate	Ion crosslinking and supramolecular interaction methods	Nanocrystalline cellulose	[58]
Lignin	Cellulose and lignin are co-dissolved in 1-ethyl-3-methylimidazolium acetate and then reconstructed with distilled water	Cellulose	[60]
Lignin	Bacterial cellulose is combined with coniferol dehydrogenation polymer	Bacterial cellulose	[63]
Chitin/chitosan	Two natural polyelectrolytes, chitosan and carboxymethyl cellulose solution, are mixed and crosslinked with glutaraldehyde	Carboxymethyl cellulose	[65]
Chitin/chitosan	Methylene bisacrylamide, as a crosslinking agent, is irradiated in acetic acid/aqueous solution to synthesize the interpolymer complex of chitosan and carboxymethyl cellulose	Carboxymethyl cellulose	[66]
Gelatin	The water-in-oil emulsion technique	Hydroxyethyl cellulose	[69]
Gelatin	Bacterial cellulose copolymerizes with gelatin	Bacterial cellulose	[70]

## 4. Applications of Modified Cellulose Hydrogels in the Sensing Field

Because hydrogel material is absorbent, its volume changes when the hydrogel material absorbs or adsorbs to liquid, which changes the electrical or optical properties of the sensor. Based on these property changes, the liquid concentration or other parameters can be measured directly or indirectly. According to the different parameters, hydrogel sensors can be divided into pH sensors, humidity sensors, pressure sensors, flexible sensors and so on. Studies have shown that relatively environmentally friendly production methods for the preparation of nanocellulose-based hydrogels already exist. The sensing properties of nanocellulose-based hydrogels are superior to those prepared by conventional materials in terms of mechanical properties, sensitivity, stability, recovery rate, etc., and have broad application prospects after solving the cost problem. Table 3 shows the applications of modified cellulose-based hydrogels in sensors.

### 4.1. Applications in pH Sensors

Humidity sensors are the most common devices used to perform humidity detection/measurement. The abundant hydrophilic group (-OH) in the cellulose chain gives it good water absorption and swelling properties, which makes cellulose an excellent choice for the preparation of humidity sensors.

Siripongpreda et al. [72] deposited the negatively charged polyelectrolyte CMC directly into the bacterial cellulose matrix, as an easy way to prepare re-expandable and biocompatible cellulose-based hydrogels. Due to this non-destructive method, the physical and mechanical properties of the original BC were well preserved in the resulting bacterial cellulose/CMC hydrogels. They can act as colorimetric pH sensors, with fast response and sensitivity, and can effectively monitor glucose in sweat.

Similarly, Lu et al. [64] were inspired by the natural function and responsiveness of lignin to synthesize lignin-based nanoparticles, as well as cellulose nanofibril-reinforced thermosensitive hydrogels. With the functions of thermal response and pH response, the introduced lignin-based nanoparticles imbued the thermal response hydrogel with a pH response, and could flexibly control the gelation temperature of the hydrogel, providing a new idea for the development of sensors based on lignin and soft-stimulation-responsive materials.

Jung et al. [73] successfully produced a pH-sensitive smart hydrogel by combining biopolymerized nanofibril with a polyelectrolyte complex. By adding a green citric acid crosslinking agent to the formed chitin- and cellulose-derived nanofiber polyelectrolytic complex, the resulting hydrogel had good structural stability, even in a water environment. The results showed that the cellulose-based hydrogel exhibits pH sensitivity, rapidly transforms surface charges, expands accordingly and selectively and efficiently removes ionic dyes based on pH conditions. The results indicate that biopolymer nanofiber hydrogels have considerable potential for application in industrial wastewater treatment and the recovery of various ionic materials, including ionic dyes.

Additionally, cellulose-based hydrogel sensors can be employed to simulate lung damage caused by drugs that results in pulmonary fibrosis. Saygili et al. [74] designed an optical pH sensor-integrated biomimetic microfluidic model to simulate drug-induced lung injury and the changes in acidity associated with cytotoxicity in cell culture. By using multiple layers of bacterial cellulose and methacrylic acid gels for tissue biomimetics, real-time pH changes were induced due to pro-fibrotic properties.

Hong et al. [75] successfully fabricated graphene oxide-reinforced regenerated cellulose/polyvinyl alcohol hydrogels through multiple freeze–thawing in NaOH/urea aqueous solution. The impacts of graphene oxide content on the mechanical properties, swelling properties and water content of the composite hydrogels were investigated. The experimental results demonstrated that the tensile strength and elongation at break of the material were notably enhanced after the addition of graphene oxide. Meanwhile, since graphene oxide is pH sensitive, the higher the pH value is, the higher the swelling ratio is. These hydrogels can serve as important materials for application in biomedical engineering and sensors.

### 4.2. Applications in Humidity Sensors

As a crucial environmental factor, humidity sensors have been extensively utilized in numerous fields, such as daily life, industrial production, agricultural production, construction engineering, biomedicine and environmental protection [76]. Due to their distinctive three-dimensional network structure, hydrogels contain a considerable number of hydrophilic and hydrophobic groups, and possess the capability to absorb and release water. There are numerous hydrophobic groups and hydrophilic residues in the natural three-dimensional network nanostructure of cellulose hydrogels, which guarantees that cellulose hydrogels can rapidly absorb and retain a large quantity of water [77]. In addition, cellulosic hydrogels have good biocompatibility, and can be used as humidity sensors in different fields.

Shi et al. [78] extracted cellulose tubers from cotton stalks under mild conditions by a one-step acid blisterable pretreatment. The hemicellulose and lignin were dissolved in lithium bromide solution, and the cellulose hydrogel was coated on the end face of the optical fiber. A fiber relative humidity sensor was prepared by using the cellulose hydrogel as a water-sensitive material. The sensitivity and stability of the sensor were studied. The results showed that the sensor can be applied to all kinds of felts, especially in high magnetic fields.

Han et al. [79] found that both graphene oxide and citral could be loaded into acrylic/bagasse cellulose and had strong hydrogen bonding with hydrogels. Therefore, they used cold plasma-induced acrylic/bagasse cellulose (AA/BC) porous hydrogel (CP), in combination with graphene oxide (GO) and embedding, to develop a novel hydrogel humidity sensor for antibacterial and smart fruit preservation. When the concentration of the GO compound was 1.0 mg/mL and the test frequency was 1 kHz, the humidity response of the acrylic/bagasse cellulose/GO (AA/BC/GO) was the highest, and it also had high conductivity. In addition, the new high-performance hydrogel humidity sensor also showed good sensitivity and durability in continuous and cyclic humidity tests, and can be used as a smart preservative in the field of food.

In addition, because hydroxypropyl methylcellulose (HPMC) is a highly water-soluble material, the hydrogel formed by HPMC swelling in water is a polymer network system with a crosslinked network structure; if exposed to the water environment, it can absorb a lot of water. Therefore, Li et al. [80] designed a relative humidity sensor based on HPMC hydrogel film. The sensor had a humidity response of 40–99% and a response time of 2.25 s, meaning that this film can be used as an excellent humidity-sensitive material for other humidity sensors. It provides new characteristics and great application potential for optical fiber sensors in biomedicine, petrochemical, food safety, environmental protection and other fields.

Fu et al. [81] used physical and chemical pretreatment and ultrasonic treatment of p-toluene sulfonic acid to promote the extraction of lignin fiber from lignocellulosic biomass, and the preparation of cellulose-based hydrogels with lignin fiber. The hydrogel was analyzed and the results showed that the hydrogel could be used as a sensor to monitor environmental humidity, with high sensitivity and good repeatability in a humidity range of 45–80%. At the same time, the study provided a sustainable method for the stabilization of lignocellulosic biomass.

### 4.3. Applications in Pressure/Strain Sensors

A pressure/strain sensor is a device that detects pressure and converts it into a usable electrical signal [82]. It measures the pressure and informs the control unit of the intensity and direction of the applied force. In recent years, a number of innovative cellulose hydrogel sensors have been developed, which possess remarkable wearable motion detection capabilities, along with high sensitivity and long durability.

**Figure 4:**
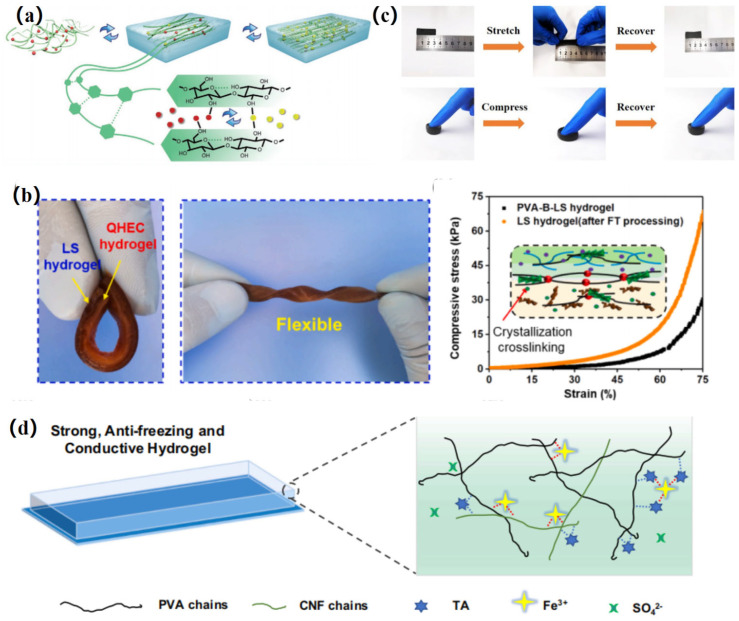
(**a**) State-transition microstructure of cellulose [83]; (**b**) photographs of bendable and flexible double-layer hydrogels and compressive stress–strain curves before and after treatment [84]; (**c**) tensile and compressive properties of hydrogel sensors [85]; (**d**) diagram of hydrogel synthesis [87].

Zhou et al. [83] designed and prepared cellulose hydrogels that can switch between fluid, brittle and rigid states by conducting Ca^2+^/Zn^2+^ ion exchange at room temperature. Figure 4a shows the microstructure of the state-converted cellulose. The high content of Ca^2+^ ions forms a rigid hydrogel with good compressive strength (about 2.2 MPa) through a coordination crosslinking network, while the Zn^2+^ ions keep the cellulose (Sol-L-Zn^2+^) in a fluid state by eliminating the connections between cellulose molecules, thus obtaining injectable and healing hydrogels. The hydrogel is a promising candidate for biomimetic and multifunctional sensors that can effectively monitor slight flexion and pressure changes in the finger site.

Since mechanical adaptability, excellent wear resistance, application stability and self-powered sensing characteristics are important requirements for hydrogel strain sensors, Wang et al. [84] fabricated hydrogels with adhesion, mechanical strength and electrical conductivity through the use of sodium lactate (LS), borax and quaternary ammonium hydroxyethyl cellulose (QHEC), for use in wearable bioelectrodes and self-powered sensors. The synergy of negative LS (−) particles and positive QHEC (+) particles gives the hydrogels good self-powered sensing ability, due to the free ion directional motion triggered by external mechanical stimulation. The hydrogel showed strong mechanical strength and self-adhesion, with a Young’s modulus of 101.3 kPa and a bond strength of 2.2 kPa. Figure 4b shows the flexible and flexible duplex hydrogel, and the compressive stress–strain curve of the hydrogel before and after treatment.

Li et al. [85] successfully prepared flexible pressure-sensitive hydrogels by using lignin as a conductive component and dispersing it uniformly in a hydrogel matrix composed of polyvinyl alcohol, carboxymethyl chitosan and CNF. Thanks to the presence of lignin-derived carbon, the hydrogel exhibits deformation-sensitive electrical conductivity, and is suitable for making flexible strain and pressure sensors for detecting human movements, such as elbow bending, finger flexion and palm grip. The material has excellent mechanical properties, with a tensile strength of 133 kPa and a compressive stress of 37.7 kPa. A picture of the hydrogel’s stretching and compression is shown in Figure 4c.

Fu et al. [86] prepared nanocomposite hydrogels through TEMPO-oxidation of nanocelluloses, gelatin and Fe^3+^. The hydrogels have Schiff base complexes, hydrogen bonds and metal coordination bonds. The compression stress of the hydrogels reaches 1310 kPa, and the hydrogels have self-healing ability and electrical conductivity. The electronic skin prepared with them can accurately recognize subtle body movements, and is recyclable.

In order to overcome the problems of weak freezing resistance, poor mechanical properties, time-consuming processes and a large amount of chemicals consumed by ionic conductive hydrogels manufactured by the traditional impregnation method, Hu et al. [87] successfully prepared a hydrogel sensor with multiple physical enhancement, anti-freezing properties and ionic conductivity by using tannic acid as a crosslinking agent under the condition of low electrolyte concentration. In this study, glycerol was employed as an anti-freeze component, and the mechanical strength of the hydrogel was significantly enhanced by establishing hydrogen bonding with polyvinyl alcohol and CNF, and forming coordination with cations in the electrolyte (such as Fe^3+^). In addition, this hydrogel is used in strain sensors, which can effectively monitor human movement. Figure 4d is a schematic diagram of the hydrogel synthesis.

In addition, Huang et al. [88] designed another organic PVA-CNF hydrogel, prepared by a one-pot method using dimethyl sulfoxide (DMSO), or a water binary solvent method using CNF and polyvinyl alcohol as raw materials. To enhance the performance of ionic conductive hydrogels, PVA-CNF hydrogels with different concentrations of CNF and Al^3+^ ions were fabricated. These hydrogels show outstanding strain response and high ionic conductivity, along with excellent stability and reliability.

### 4.4. Applications in Flexible Sensors

At present, due to their light weight, high sensitivity and good stretchability and flexibility, flexible wearable sensors have received extensive attention [89]. In the research field of flexible sensors, hydrogel sensors based on cellulose have become an important research direction in wearable devices. When the hydrogel sensor is subjected to external force, it will deform, resulting in a change in resistance. The measuring factor can accurately detect weak signals of human movement and provide comfortable wearability, as well as good repeatability and fatigue resistance.

**Figure 5:**
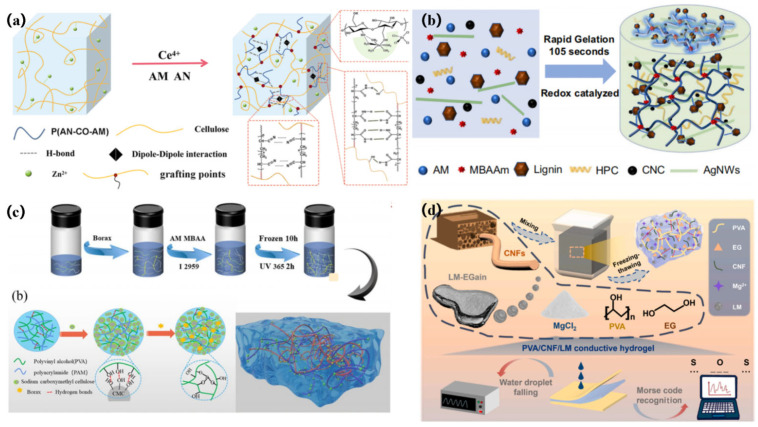
(**a**) Hydrogel preparation diagram [90]; (**b**) copolymerization of lignin [61]; (**c**) synthesis method of hydrogels and dual-network synthesis mechanism [94]; (**d**) schematic diagram of conductive hydrogel [95].

Chen’s team [90] designed and implemented a flexible cellulose-based conductive hydrogel sensor. Figure 5a is a schematic of the preparation the hydrogel. By employing zinc nitrate as a catalyst, and with the assistance of zinc chloride, they successfully grafted the copolymer of acrylonitrile and acrylamide onto the cellulose molecular chain, thereby synthesizing this novel flexible cellulose hydrogel material. The material shows outstanding extensibility properties, including excellent tensile strength, high elasticity and toughness, as well as good electrical conductivity. In addition, this hydrogel also has excellent fatigue resistance and anti-freeze effects, meaning that it can efficiently and stably detect human movement.

In addition, Jing et al. [91] prepared a PVA/CNF hydrogel containing a double-crosslinked network for the manufacture of highly transparent, stretchable and self-healing pressure and strain sensors. The hydrogel contains dynamic borate ester bonds, metal–carboxylic acid coordination bonds and hydrogen bonds. The presence of these bonds enables the hydrogel to exhibit better dimensional stability, mechanical properties, flexibility and self-healing ability compared to traditional borate ester bond materials, and the hydrogel also demonstrates good biocompatibility with human fibroblasts. This new hydrogel and its sensors not only show great potential in the field of electronic skin, personal health monitoring and wearable technology, but may also advance the development of transparent smart skin sensors.

Similarly, Song et al. [92] used a novel dynamic redox system composed of sodium lignosulfonate/Fe^3+^ to successfully prepare a conductive hydrogel, using acrylic acid and sodium CMC as substrates, at room temperature. The system significantly speeds up the polymerization process of monomers (CMC and AA) by activating potassium persulfate (KPS), which generates a large number of free radicals, while releasing a large amount of heat. In addition, the introduction of Fe^3+^ can achieve dynamic double-crosslinking of polymer chains, giving hydrogels excellent mechanical properties, high ionic conductivity, good sensing sensitivity and electrical self-healing function. The material can serve as a flexible sensor for detecting a wide variety of human movements, covering the extensive movements of fingers, elbows, wrists and knees, as well as subtle movements like swallowing.

Using 4-formylbenzoylboric acid as a crosslinking agent, Qiu et al. [93] crosslinked polyvinyl alcohol and polyvinylimide to form a fast-self-healing hydrogel network containing borate and imide bonds. In addition, they introduced polypyridine-modified CNF (PPy@CNF) to successfully prepare a cellulose-based hydrogel with excellent mechanical and electrical properties. After being packaged, the material can be used as a flexible sensor for human body monitoring, which can effectively detect the movement of various parts of the human body.

Similarly, in order to be able to develop a flexible sensor with excellent mechanical properties, self-adhesion and biocompatibility, Chen et al. [61] developed a semi-interpermeable polymer network structure using lignin-grafted polyacrylamide/hydroxypropyl cellulose hydrogels. Figure 5b shows the copolymerization scheme of lignin. This polymer network structure, compared with polyacrylamide-based hydrogels, has special skin adhesion and tensile properties. At the same time, due to the addition of conductive additives such as silver nanowires and carbon nanocages, the hydrogel also has excellent conductivity.

Xu et al. [94] designed a double-mesh interpenetrating structure by mixing polyvinyl alcohol and polyacrylamide to form hydrogen bonds, and added sodium CMC and borax to enhance the mechanical properties of the double-mesh polymer hydrogel and improve its tear resistance and adhesion. The test results showed that the cellulose-based hydrogel can be used as a flexible sensor to monitor human motion, temperature and humidity. Figure 5c illustrates the hydrogel synthesis method and the interaction penetration network mechanism.

Hang et al. [95] developed an exploitable conductive hydrogel sensor with high toughness and adaptability, and constructed a conductive composite hydrogel based on high-performance polyvinyl alcohol with the assistance of green CNF, magnesium chloride, ethylene glycol and liquid metal, as shown in Figure 5d. The synergistic effect between the green CNF and the liquid metal enhances the network structure inside the recyclable hydrogel, which results in excellent tensile strength and elongation at break. The compressive strength at 80% strain is 4.04 MPa. In addition, a conductive network composed of magnesium chloride and liquid metal gives the hydrogel good electrical conductivity.

Ling et al. [96], using dialdehyde carboxymethyl cellulose (DCMC), chitosan (CS), poly (acrylic acid) (PAA) and aluminum (Al^3+^) ions, constructed a highly stretchable, sensitive and multifunctional polysaccharide-based dual-network hydrogel sensor. The obtained DCMC/CS/PAA (DCP) composite hydrogel showed strong mechanical strength, repeatable underwater adhesion and good self-healing properties for animal tissues; could sensitively monitor human movements, including finger bending, smiling and wrist pulse; and could stably detect human movements underwater. This work is expected to provide new strategies for the design of high-performance smart sensors, especially for applications in wet and underwater environments.

Hao et al. [97] prepared a kind of intelligent temperature sensor based on ionized water gel. Based on the temperature-dependent self-association interaction of methacrylate sulfobetaine polymer chains and the incorporation of thermosensitive tetramethylpiperidine oxide-oxidized cellulose/polyaniline nanofibers/polyaniline nanofibers in a glycerin–water binary solvent system, the hydrogel has anti-freeze properties. The time signal recognition and local temperature detection of this sensor are successful.

In addition, the researchers also gave more properties to flexible sensors, such as attaching zinc oxide to cellulose, and adding sodium alginate by ultrasonic dispersion technology, creating a multiple-crosslinking network, giving the cellulose hydrogel sensor an antibacterial function [98]. The sensor has a high tensile performance of more than 2000% strain, meaning that it can not only act as a flexible sensor for human health detection, but also serve as a sound detection device to detect speech and breathing. It provides new possibilities for human health detection.

**Table 3:** Applications of modified cellulose-based hydrogels in sensors.

Type of Application	Modified	Principle	Ref.
pH sensor	Polyelectrolyte CMC is deposited directly into the bacterial cellulose matrix	A universal pH indicator or glucose oxidase is added to act as a colorimetric pH or glucose sensor, respectively	[72]
pH Sensor	Lignin-based nanoparticles and cellulose nanofibril	The introduced lignin-based nanoparticles determine the pH response of heat-responsive hydrogels	[64]
pH sensor	Biopolymerized chitin and nanofibril	The addition of green citric acid crosslinkers enables rapid surface charge conversion, consequent expansion and selective and efficient removal of ionic dyes, depending on pH conditions	[73]
pH sensor	Repeated freeze–thaw method in aqueous NaOH/urea solution	GO-enhanced regenerated cellulose/PVA ternary hydrogel has pH sensitivity	[75]
Humidity sensor	Lignin modification	A fiber optic relative humidity sensor is prepared by using cellulose hydrogel as a water-sensitive material	[78]
Humidity sensor	Both graphene oxide and citral are loaded into acrylic/bagasse cellulose and have strong hydrogen bonding with hydrogels	The added graphene oxide is humidity-sensitive	[79]
Pressure sensor	By Ca^2+^/Zn^2+^ ion exchange at room temperature, three states of fluid, brittle and rigid cellulose hydrogels are designed and prepared	The Ca^2+^ ion forms good compressive strength through a coordination crosslinking network, while the Zn^2+^ ion transforms cellulose (Sol-L-Zn^2+^) into a fluid state by eliminating the connections between cellulose molecules	[83]
Pressure sensors	Lignin modification	The free-ion-directed motion induced by external mechanical stimulation and the synergistic effect of negative LS (−) particles and positive QHEC (+) particles give the hydrogel good self-energy sensing ability	[84]
Pressure sensor	Lignin modification	Lignin is used as a conductive filler	[85]
Flexible sensor	Acrylonitrile and acrylamide copolymers are grafted onto the cellulose chain in the presence of zinc chloride, with zinc nitrate as an initiator	The cellulose hydrogels prepared have superstretchability, excellent tensile strength, high elasticity, good toughness and electrical conductivity, as well as fatigue resistance due to the presence of multiple hydrogen bond interactions in both dipole–dipole interactions and the hydrogel networks.	[90]
Flexible sensor	Lignin modification	Through a new dynamic REDOX system composed of sodium lignesulfonate/Fe^3+^, the introduction of Fe^3+^ can dynamically double-crosslink polymer chains, giving hydrogels good mechanical properties, better ionic conductivity, good sensing sensitivity and electrical self-repair.	[92]
Flexible Sensor	Introduction of polypyrrole-modified cellulose nanofibers	As a crosslinking agent, 4-formylphenylboric acid is used to crosslink polyvinyl alcohol and polyethylenimine, and a hydrogel network with borate ester bonds and imine bonds is constructed	[93]
Flexible sensor	Lignin graft modification	Lignin-grafted polyacrylamide/hydroxypropyl cellulose hydrogels have special skin adhesion and tensile properties	[61]

## 5. Challenges and Future Perspectives

As a new type of green material, cellulose-based hydrogels have been widely reported in recent years. In view of their excellent performance and green and renewable environmental characteristics, they have been used as humidity, pressure and pH sensors. In this review, the research progress regarding the physical and chemical modification of cellulose, cellulose-based hydrogels modified by biomass and modified cellulose hydrogels in the field of sensing were reviewed. However, relatively speaking, the application of this composite material is still at an early stage, and has not been deeply and systematically researched and developed, nor has it been put into industrial large-scale production. In addition, there are still some problems that need to be solved, such as the complicated process costs of modified cellulose, the mechanical properties of cellulose-based hydrogel sensors after repeated use and the sensitivity of sensors. Based on these circumstances, we need to further discuss the pretreatment of cellulose; optimizing its adjustable and self-healing properties, performance stability and recycling practicability are key to the actual application process. In the future, modified cellulose-based hydrogels have broad development prospects, and researchers can explore their applications in multifunctional sensors, such as composite sensors that integrate multiple sensing functions such as temperature, humidity and pressure, focusing on the development of integrated systems with multiple functions such as sensing, energy storage and self-healing. In addition, researchers can also explore their potential applications in the aerospace and automotive industries, and other fields of multifunctional integration. With the deepening of research and technological progress, modified cellulose-based hydrogels will contribute more to the development of human society in the field of sensing.

## Data Availability

No new data were created or analyzed in this study.
